# Clinically Translatable Phosphonated Silica Microspheres for Selective Internal Radiation Therapy of Hepatocellular Carcinoma

**DOI:** 10.1002/smsc.202300035

**Published:** 2023-08-22

**Authors:** Yi Zhou, Jianxian Ge, Yun Gao, Zhe Yang, Mohammad Javad Afshari, Can Chen, Manran Wu, Lei Chen, Shuwang Wu, Guangxin Duan, Jianfeng Zeng, Mingyuan Gao

**Affiliations:** ^1^ Center for Molecular Imaging and Nuclear Medicine, State Key Laboratory of Radiation Medicine and Protection, School for Radiological and Interdisciplinary Sciences (RAD-X) Soochow University Collaborative Innovation Center of Radiological Medicine of Jiangsu Higher Education Institutions Suzhou 215123 China; ^2^ Suzhou Xinying Biomedical Technology Co. Ltd. Suzhou 215000 China

**Keywords:** phosphonated silica microspheres, radioactive microspheres, selective internal radiation therapy

## Abstract

Selective internal radiation therapy (SIRT) is used as a locoregional therapy for hepatocellular carcinoma (HCC) to delay the progression of HCC. Thus far, various kinds of radioactive microspheres with different materials and radionuclides have been developed to be utilized in SIRT, but they remain unsatisfactory in some ways. Herein, using a facile method, phosphonated silica microspheres (PSMs) are prepared and further radiolabeled with ^177^Lu in a rapid and effective manner. The results suggest that the radiolabeling efficiency is higher than 99%, and the specific activity of the product is 10 kBq per microsphere. ^177^Lu‐PSM exhibits outstanding in vivo radiolabeling stability, excellent antitumor activity, and single‐photon‐emission computed tomography–computed tomography (SPECT/CT) imaging capability. In addition, it is shown that PSM can be produced in large quantities at a relatively low cost. The results indicate that ^177^Lu‐PSM might hold considerable potential for translation to clinical applications.

## Graphical abstract

Phosphonated silica microspheres (PSM) are prepared by a facile method to be radiolabeled with ^177^Lu in a rapid and effective manner. The prepared ^177^Lu‐PSM exhibits excellent antitumor activity and SPECT/CT imaging capability, which indicate that ^177^Lu‐PSM might hold considerable potential for translation to clinical applications.

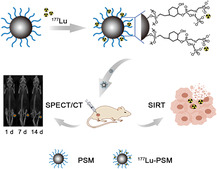

## 1 Introduction

Selective internal radiation therapy (SIRT), also called radioembolization, has attracted growing attention over the past decades.^[^ ^1^ ^]^ The rationale behind SIRT lies in the fact that the hepatic artery, which supplies blood to tumor tissue in the liver, has a higher density compared to the portal vein, which primarily supplies blood to normal liver tissue.^[^ ^2^ ^]^ This unique anatomical characteristic allows SIRT to target the tumor directly by delivering radioactive microspheres into the artery that supplies it.^[^ ^3^ ^]^ Subsequently, the beta rays emitted by the radionuclides on microspheres can destroy tumor cells located in their close vicinity. In comparison with external radiation therapy, SIRT can effectively avoid unwanted damage to normal tissues, which is usually induced by high‐energy ionizing radiation.^[2b]^ Due to this distinctive feature, SIRT is widely used in palliative or downstaging treatments of hepatocellular carcinoma (HCC).^[^ ^4^ ^]^

Among the various kinds of radioactive microspheres developed for SIRT, three of them (i.e., TheraSphere, SIR‐Sphere, and QuiremSphere) have become commercially available for treating HCC.^[^ ^5^ ^]^ TheraSphere is a kind of yttrium‐90 (^90^Y)‐labeled glass microsphere with a diameter range of 20–30 μm. As the first step in preparing the microspheres, nonradioactive ^89^Y_2_O_3_ was fused to the glass. Subsequently, the resulting ^89^Y‐glass microspheres were activated to ^90^Y‐glass microspheres by neutron beam irradiation.^[^ ^6^ ^]^ The product exhibited high specific activity (i.e., 2500 Bq microsphere^−1^), such that ≈1.2–8 million can achieve a suitable therapeutic dose for SIRT.[^1^, ^7^] However, the method of production is rather complex and time‐consuming, such that high temperature is needed to form microspheres and long‐term activation is necessary to obtain radioactive agents with adequate radioactivity.^[^ ^6^, ^8^ ^]^ In contrast, the preparation of SIR‐Sphere is performed through ion‐exchange absorption of ^90^Y by resin microspheres, which is relatively less complicated and results in ^90^Y‐resin microspheres whose sizes fall within the range of 20–60 μm.^[^ ^9^ ^]^ However, their low specific activity (i.e., 50 Bq microsphere^−1^) necessitates an approximate number of 40–80 million microspheres to provide adequate SIRT doses for treating humans.[^1^, ^10^] Notably, such a large number of microspheres in the hepatic artery might tend to cause reflux, which in turn exposes normal tissues to unwanted ionizing radiation and thus provides the lesion areas with insufficient therapeutic doses.^[^ ^11^ ^]^ Different from the two agents mentioned above, QuiremSphere takes advantage of holmium‐166 (^166^Ho) to radiolabel poly(l‐lactic acid) (PLLA) microspheres, forming ^166^Ho‐PLLA microspheres in the 15–60 μm size range. To this end, PLLA microspheres containing ^165^Ho are activated to ^166^Ho‐PLLA microspheres utilizing a nuclear reactor, in which a relatively low specific activity (i.e., 450 Bq microsphere^−1^) is achieved.^[^ ^12^ ^]^ Nevertheless, the structure of PLLA microspheres is prone to be destroyed by high temperature induced during neutron activation.^[^ ^13^ ^]^

As indicated earlier, commercially available agents have faced certain challenges that remain to be addressed. Moreover, taking the half life of radioisotopes into consideration, the products should be transported and administered shortly after their production. This might severely limit their applications for situations in which there is a large distance between the company and the destination. Ideally, radioactive microspheres should take advantage of a straightforward preparation method, low preparation cost, and stability in physicochemical properties during the storage period and sterilization courses. Herein, we developed novel radioactive microspheres through a straightforward method in which the product could be stored for a long time and the isotope was able to be quickly and effectively labeled prior to SIRT. To this end, silica microspheres (SMs) were used due to the stability of their physiochemical properties as well as their excellent biocompatibility for in vivo applications. Phosphonate siloxane prepared by β‐(3,4‐epoxycyclohexane) ethyl trimethoxy silane (EHTME) and amino trimethylene phosphonic acid (ATMP) was used to functionalize the microspheres. The phosphonate groups on the surface of the microspheres were utilized to chelate radionuclides. Lutetium‐177 (^177^Lu), which is a β and γ emitter, was chosen as the radionuclide to endow the microspheres with the capability of destroying tumor cells by β particles and visualizing their biodistribution by γ rays.^[^ ^14^ ^]^ More importantly, the radiolabeling of microspheres can be performed just before usage, which can avoid the decrease in specific activity caused by decay in transportation, thus broadening the prospects for the application of SIRT in developing countries/areas where the availability of nuclear facilities is restricted.^[^ ^15^ ^]^

## 2 Results and Discussion

**Scheme 1:**
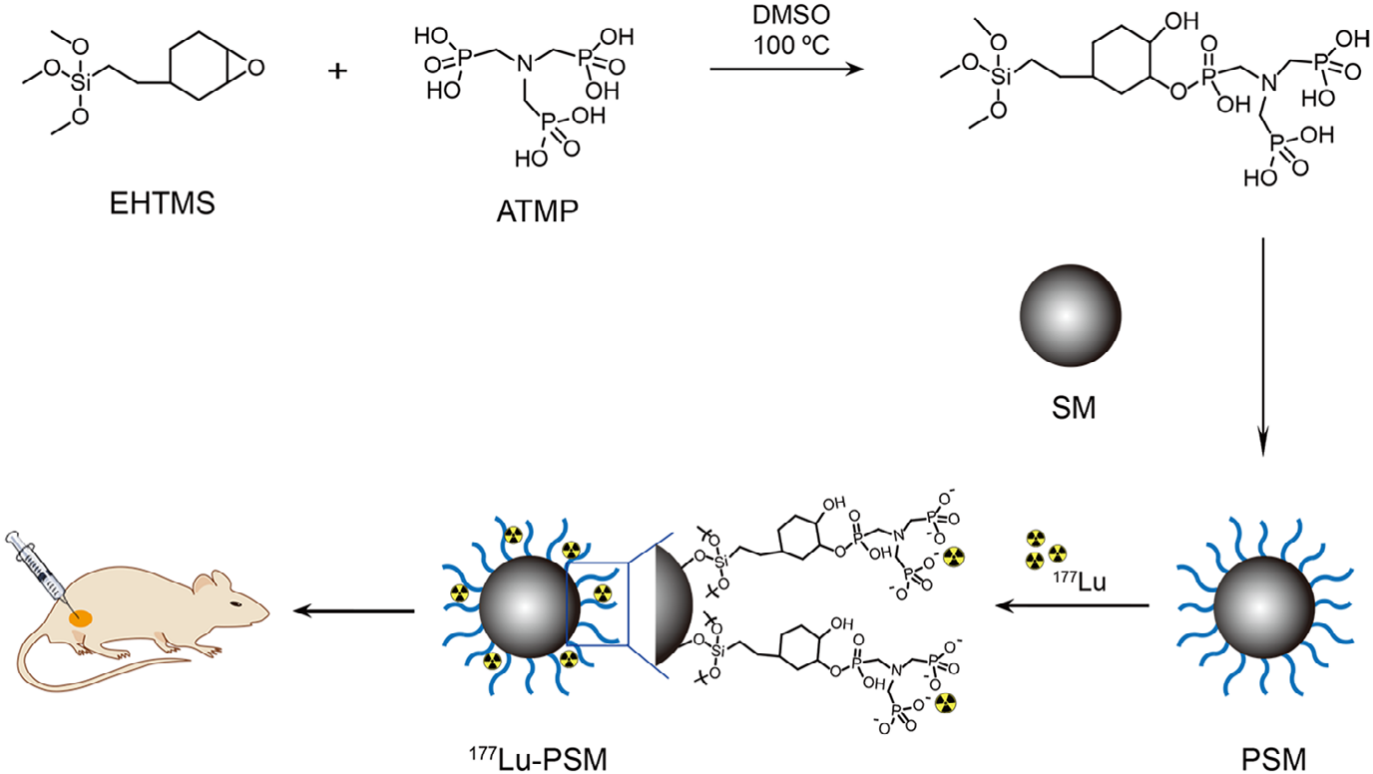
Schematic illustration of the method with which clinically translatable PSMs and ^177^Lu‐PSM were prepared and employed.

**Figure 1:**
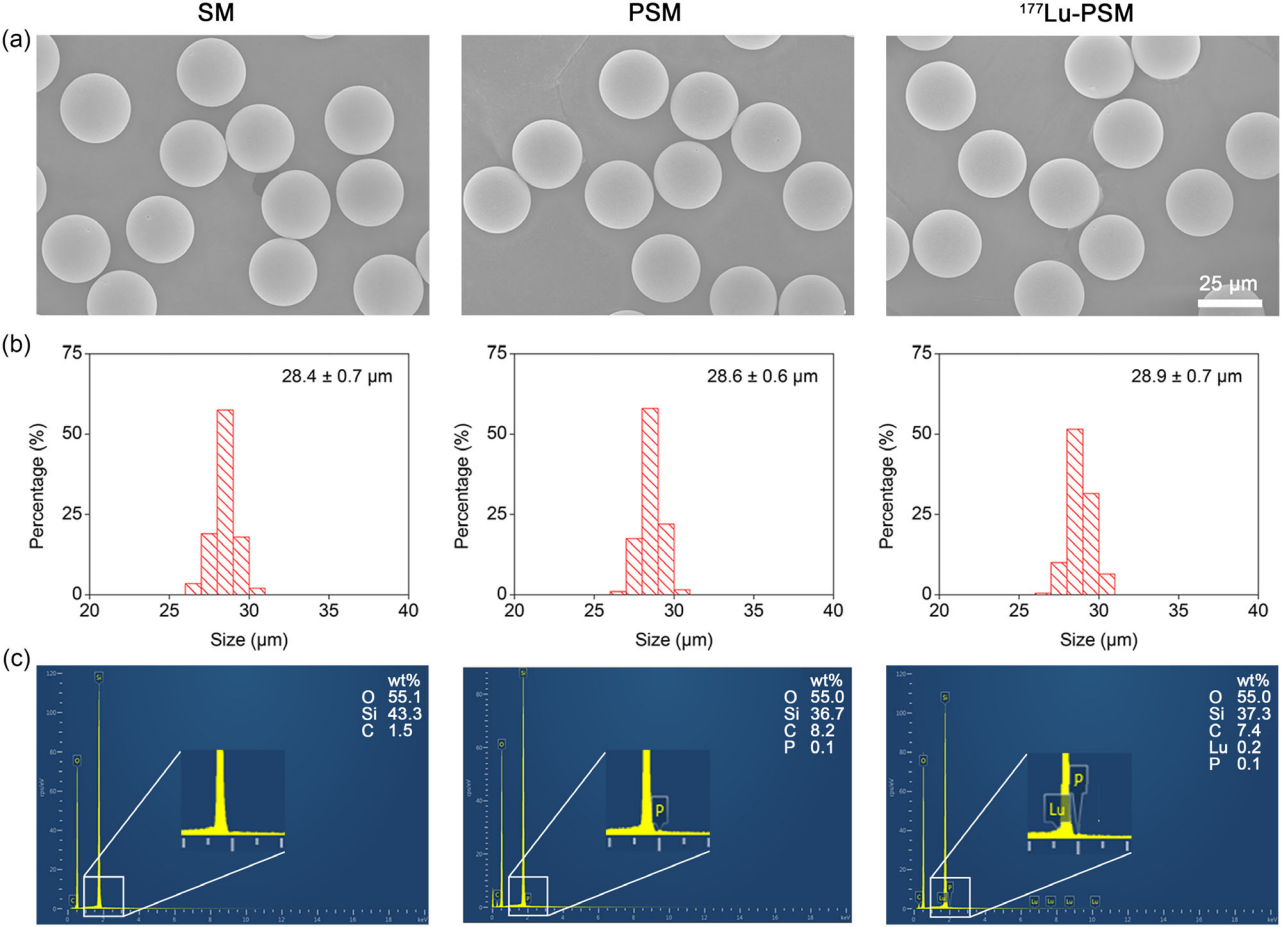
a) SEM images of SiO_2_ microspheres (SM), PSMs, and ^175^Lu‐PSM. b) Size distribution histogram of SM, PSM, and ^175^Lu‐PSM. c) EDX spectrum data of SM, PSM, and ^175^Lu‐PSM.

As shown in **Scheme** **1**, the phosphonated siloxane was first synthesized by the ring‐opening reaction between β‐(3,4‐epoxycyclohexane) ethyl trimethoxy silanea (EHTMS) and amino trimethylene phosphonic acid (ATMP). Then, phosphonated silica microspheres (PSMs) were obtained by cohydrolysis of phosphonated siloxane and tetraethyl orthosilicate (TEOS) onto hydrochloric acid‐activated SMs. The scanning electron microscopy (SEM) image shown in **Figure** **1**a revealed that the starting SMs were perfectly spherical in shape with an average diameter of 28.4 μm. Upon phosphonate functionalization, the shape, size, and size distribution of the resulting PSMs remained unchanged (Figure 1b). In addition, as seen in Figure 1c, the emergence of the phosphorus characteristic peak (denoted as P) on the energy‐dispersive X‐ray spectroscopy (EDS) spectrum demonstrated the successful surface modification of SM with phosphonate groups. Subsequently, to demonstrate the capability of PSM to chelate radiometal ions, nonradionuclides lutetium‐175 (^175^Lu) was first adopted to prepare ^175^Lu‐labeled PSM (^175^Lu‐PSM) by mixing ^175^LuCl_3_ solution with PSM in sodium acetate buffer (pH = 4.6). Drawing a comparison between the SEM image, size distribution histogram and EDS spectrum of the labeled product with those of its mothers (Figure 1b) indicated the successful occurrence of the labeling process. To further verify this statement, X‐ray photoelectron spectroscopy (XPS) was performed. As shown in Figure S1 (Supporting Information), the high‐resolution P 2p and Lu 4 d spectra emerged in the corresponding curves of PSM and ^175^Lu‐PSM, respectively. Accordingly, Table S1 (Supporting Information) lists the atomic percent of surface elements (i.e., Si, O, C, N, P, Lu) for SM, PSM, and ^177^Lu‐PSM. The consistency of the results with those of EDS served as further proof for the correct occurrence of the designed preparation route.

**Figure 2:**
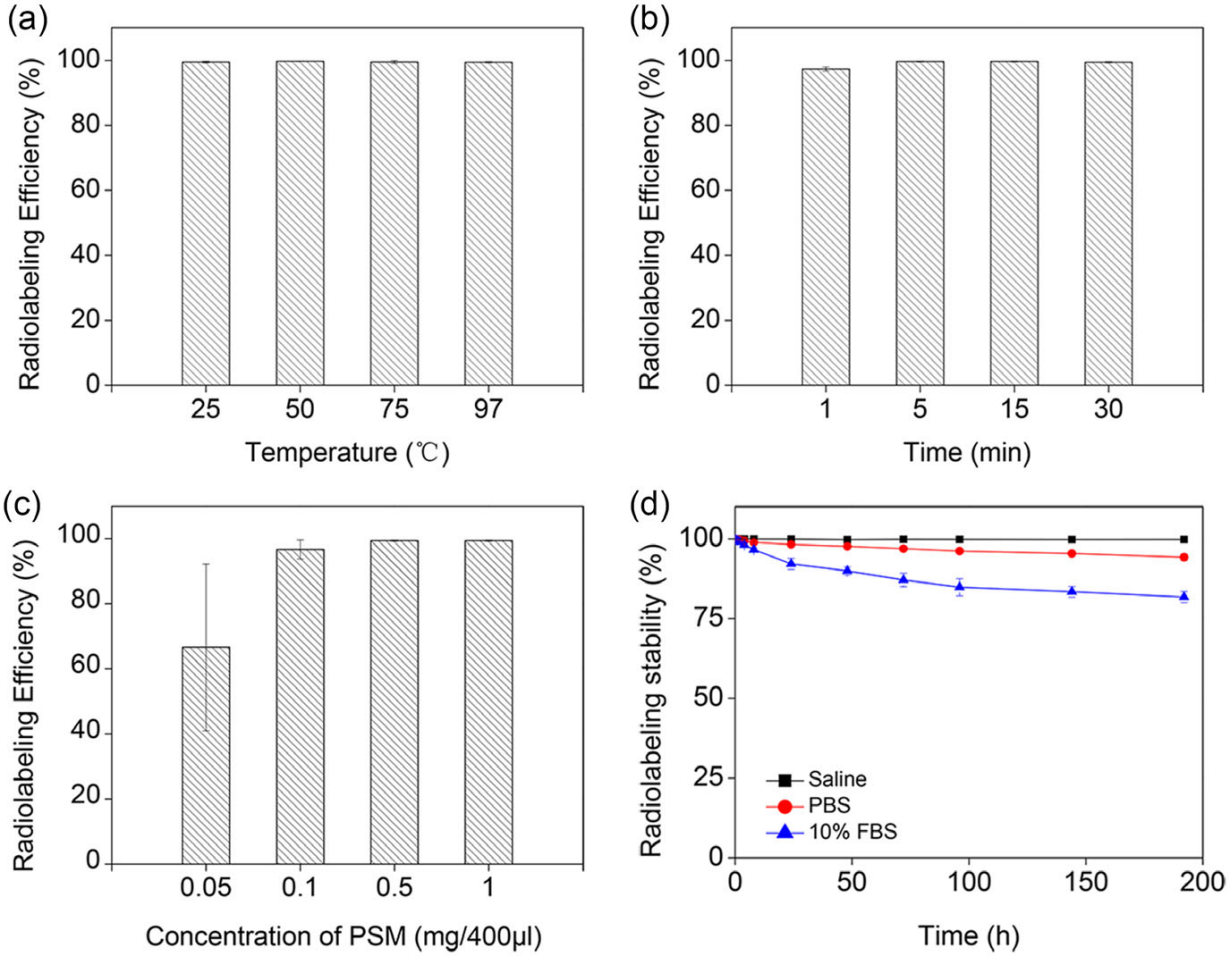
a–c) Radiolabeling efficiency of ^177^Lu‐PSM at different temperatures (a), times (b), and PSM concentrations (c). d) Radiolabeling stability of ^177^Lu‐PSM in saline, PBS, and 10% FBS. The error bars represent the standard deviation (SD) of three replicates.

To investigate the efficiency and stability of ^177^Lu radiolabeling, corresponding experiments were conducted in 400 μL of sodium acetate buffer solution (0.2 m, pH = 4.56). As illustrated in **Figure** **2**a, the radiolabeling efficiency was almost independent of temperature for a range of 25–97 °C. The radiolabeling efficiency was 99.4% ± 0.3% at 25 °C, which is almost the same as that at 97 °C (i.e., 99.4% ± 0.2%). Moreover, the radiolabeling efficiency as a function of reaction time is given in Figure 2b. The results suggested that the radiolabeling reaction took place rapidly (within 1 min) with an efficiency value of 97.3% ± 0.6%, which was almost the same for longer reaction times up to 30 min. Furthermore, the influence of PSM concentration on radiolabeling efficiency was investigated. As shown in Figure 2c, employing a concentration value as low as 0.1 mg/400 μL could give rise to a high radiolabeling efficiency of 96.7% ± 3.0%. Taking the obtained results into consideration, radioactive SMs with a high radiolabeling efficiency at room temperature, short radiolabeling time, and low concentration of matrix could be achieved through a universal and straightforward method. Notably, as shown in **Table** **1**, the estimated specific activity of ^177^Lu‐PSM (≈10 000 Bq microsphere^−1^) was much higher than those of the three commercially available radioactive microspheres mentioned above. After the preparation of ^177^Lu‐PSM, the radiolabeling stability was investigated in normal saline, phosphate‐buffered saline (PBS), and 10% fetal bovine serum (FBS) at room temperature. As shown in Figure 2d, the release of ^177^Lu is 0.2%, 5.8%, and 18.2% upon incubation of the product in normal saline, PBS, and 10% FBS for 192 h, respectively, which indicated its good stability in physiological media.

**Table 1:** Characteristics of commercialized radioactive microspheres and ^177^Lu‐PSM

	TheraSphere	SIR‐Spheres	QuiremSpheres	^177^Lu‐PSM
Material	Glass	Resin	PLLA	SMs
Mean diameter [μm]	20–30	20–60	15–60	30, 50, 100
Radionuclide	^90^Y	^90^Y	^166^Ho	^177^Lu
Half‐life [d]	2.7	2.7	1.1	6.7
Specific activity [Bq microsphere^−1^]	2500	50	450	10 000

**Figure 3:**
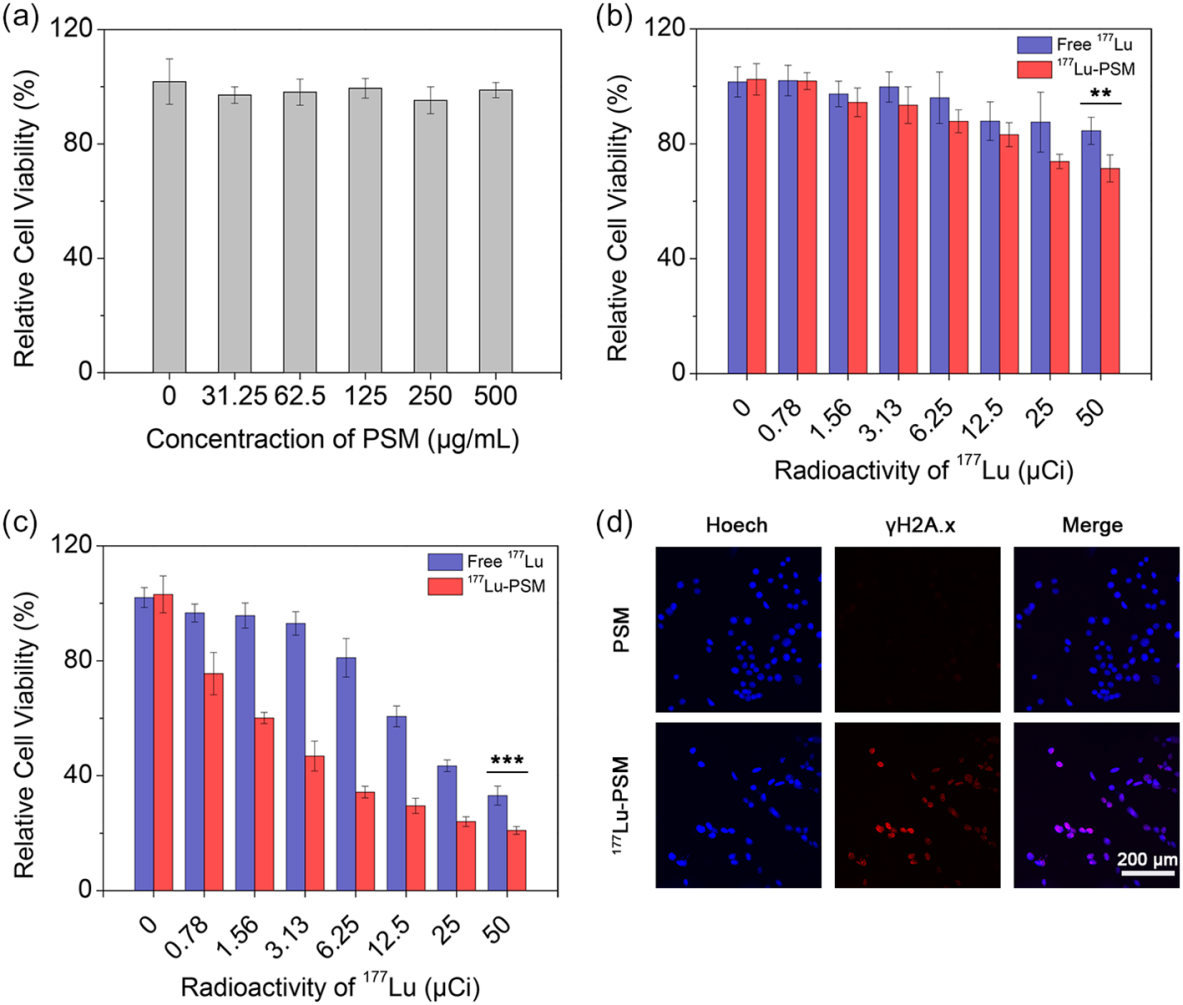
a) Relative cell viability of HepG2 cells treated with PSM at different concentrations for 24 h. b,c) Relative cell viability of HepG2 cells treated with free ^177^Lu and ^177^Lu‐PSM at different radioactivity for 24 h (b) and 48 h (c). d) Immunofluorescence of HepG2 cells treated with PSM and ^177^Lu‐PSM. The error bars represent the SD of five replicates. *P* values was calculated by Student's *t*‐test (*P < 0.05, **P < 0.01, and ***P < 0.001).

To evaluate the biosafety of PSM, different concentrations of the microspheres were incubated with HepG2 cells for 24 h. Using a cytotoxicity assay, the cell viability values were measured to be above 95.5% ± 4.7% for a concentration as high as 500 μg mL^−1^ (**Figure** **3**a). Thus, the results suggested that PSM exhibited excellent biosafety. Furthermore, a series of investigations were carried out to compare the degree to which free ^177^Lu and ^177^Lu‐PSM were able to induce cytotoxicity in HepG2 cells. To this end, the cells were incubated with radioactive agents at different doses for 24 and 48 h. As illustrated in Figure 3b,c, at a certain radioactive dosage, the cytotoxicity induced by ^177^Lu‐PSM was higher than that provided by free ^177^Lu. This might be caused by the longer separation distance between free ^177^Lu and the cells adhered to the well bottom. Indeed, the distribution of free ^177^Lu and ^177^Lu‐PSM in the medium during the incubation process is different. Free ^177^Lu tends to disperse uniformly in the medium, while ^177^Lu‐PSM has a tendency to sediment within the well due to its physical properties. This nonuniform distribution of ^177^Lu‐PSM can result in a higher concentration of radioactive microspheres in the proximity of the cells. Furthermore, considering the maximum tissue penetration range of β particles generated from ^177^Lu, which is 2.2 mm, and the height of the sample well (≈3.1 mm), it becomes evident that cells incubated with ^177^Lu‐PSM will receive more energy deposition. This is due to the close proximity of the microspheres to the cells, allowing for increased radiation doses. On the contrary, cells incubated with free ^177^Lu might be located beyond the range of the β‐particles, leading to less energy deposition and potentially lower cytotoxicity. Therefore, the radioactive microspheres, ^177^Lu‐PSM, are likely to induce higher cytotoxicity in this experiment compared to free ^177^Lu. This understanding highlights the potential advantage of using ^177^Lu‐PSM for delivering targeted radiation therapy to specific cells or tissues, increasing the therapeutic effect while reducing damage to surrounding healthy tissues. Moreover, for both 24 and 48 h incubation periods, the relative viability of HepG2 cells decreased with the enhancement in the radioactivity of ^177^Lu, indicating that the cytotoxicity was highly dependent on the radioactivity of the radioisotope. Additionally, the cytotoxicity induced by the radioactive agents was also dependent on the incubation time. Taking the relative cell viability of HepG2 cells incubated with 50 μCi ^177^Lu‐PSM as an example, the cell viability decreased from 71.4% ± 4.7% to 30.0% ± 1.4% when the time extended from 24 to 48 h. In addition, the immunofluorescence images (Figure 3d) revealed that ^177^Lu‐PSM could effectively induce damage to the DNA of HepG2 cells.

**Figure 4:**
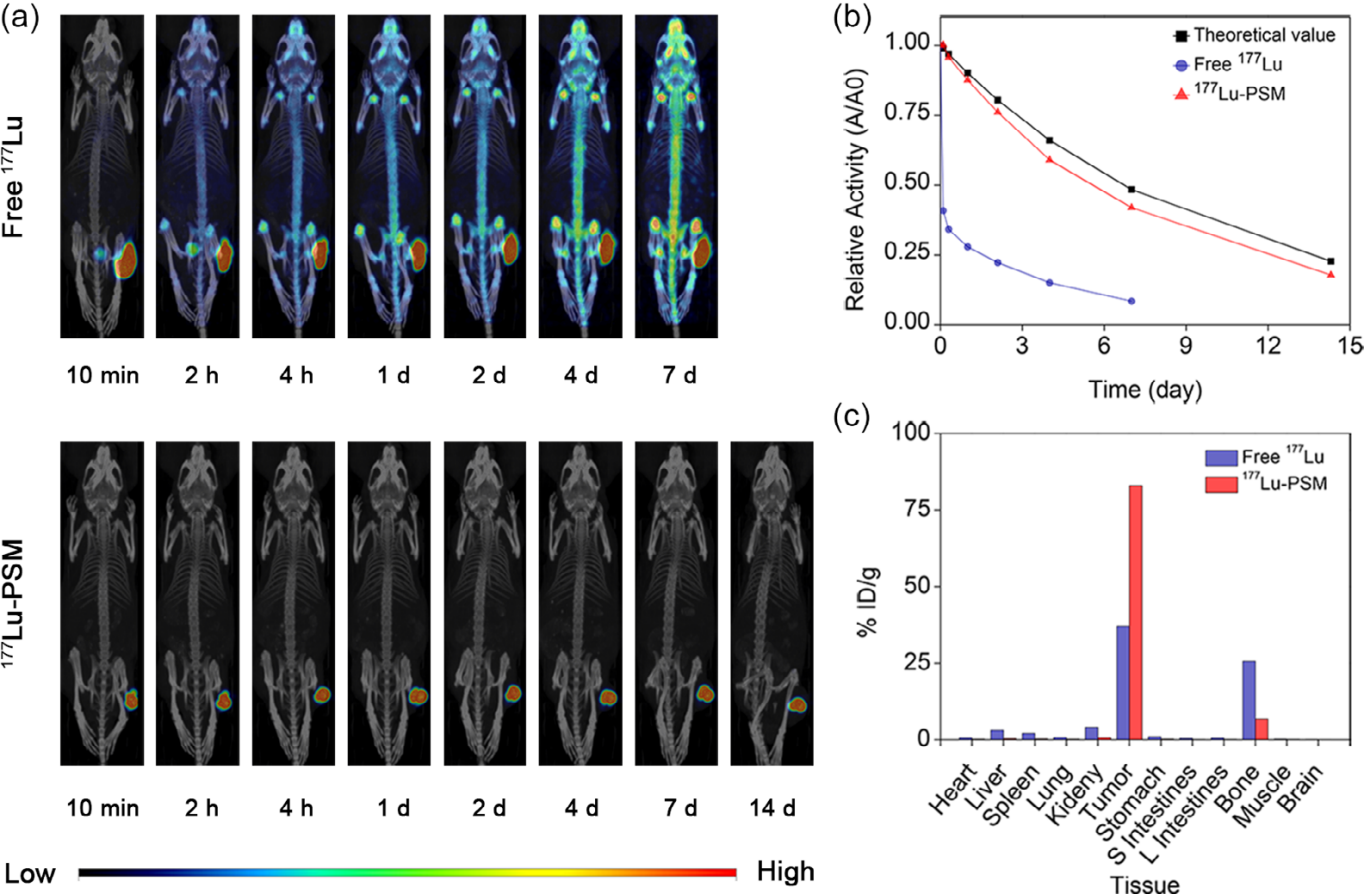
a) SPECT/CT images of HepG2‐tumor‐bearing mice intratumorally treated with free ^177^Lu (700 μCi) and ^177^Lu‐PSM (700 μCi). b) Relative activity of ^177^Lu in theoretical decay and tumorous regions after the administration of free ^177^Lu and ^177^Lu‐PSM. c) Biodistribution of ^177^Lu obtained 7 d and 14 d after treatment with free ^177^Lu and ^177^Lu‐PSM.

Encouraged by their significant biosafety and the excellent capability of tumor cell destruction, the in vivo properties of the prepared microspheres were further investigated. To evaluate the in vivo imaging capability and radiolabeling stability of ^177^Lu‐PSM, whole‐body single‐photon‐emission computed tomography–computed tomography (SPECT/CT) images of HepG2‐tumor‐bearing mice were captured upon intratumoral administration of free ^177^Lu (700 μCi) or ^177^Lu‐PSM (700 μCi). As shown in **Figure** **4**a, for the group treated with free ^177^Lu, the radioisotope was rapidly taken up by the bladder and then distributed to the whole skeleton of the mouse within 2 h. As time progressed, the ^177^Lu signal arose from the skeleton, especially the joints and spine. In contrast, in the case of mice treated with ^177^Lu‐PSM, the signal was perfectly localized in the tumor region, while no detectable signal was detected from other tissues for up to 14 d postinjection. This indicates that ^177^Lu is tightly bound to the PSM with negligible detachment within 14 days. Moreover, the temporal variation in the relative activity of the administered compounds was calculated by analyzing the signal arising from a region of interest (ROI) localized on the tumor site. The results presented in Figure 4b indicated that ^177^Lu‐PSM exhibited a relative activity value similar to that obtained from the theoretical decay curve of ^177^Lu, further demonstrating the in vivo radiolabeling stability of ^177^Lu‐PSM. Conversely, the curve for free ^177^Lu decayed quickly, and thus, its corresponding relative activity was much lower than that of the theoretical calculation.

To evaluate the biodistribution of the compounds, the animals were sacrificed at 14 d postinjection, and their major organs as well as tumor tissues were harvested. The percent injected dose per gram (% ID/g) values of the collected tissues were calculated and are illustrated in Figure 4c. As shown in the figure, the radioactivity of the ^177^Lu‐PSM group was perfectly localized in the tumor with a percent ID/g value of 83.0. Moreover, negligible values from other organs, except bone with a low percent ID/g of 6.85, could be detected. The slight accumulation of ^177^Lu in the bone can indeed be attributed to the strong osteophilic nature of lutetium. As shown in Figure 2d, a small amount of ^177^Lu was released from the ^177^Lu‐PSM after incubation in physiological medium, and this released ^177^Lu could potentially contribute to the observed uptake in the bone tissue. This consistency between the in vitro and in vivo findings reinforces the understanding that ^177^Lu‐PSM exhibits good in vivo radiolabeling stability. In contrast, the mice treated with free ^177^Lu exhibited a much lower % ID/g value for tumor (i.e., 37.0). Apart from the tumor, other organs, such as bone, kidney, liver, and spleen, could take up the element with percent ID/g values of 25.6, 4.1, 3.1, and 2.1, respectively. The higher accumulation of ^177^Lu‐PSM in the tumor and the limited uptake in other organs suggest the potential of ^177^Lu‐PSM as a promising candidate for SIRT in HCC treatment.

**Figure 5:**
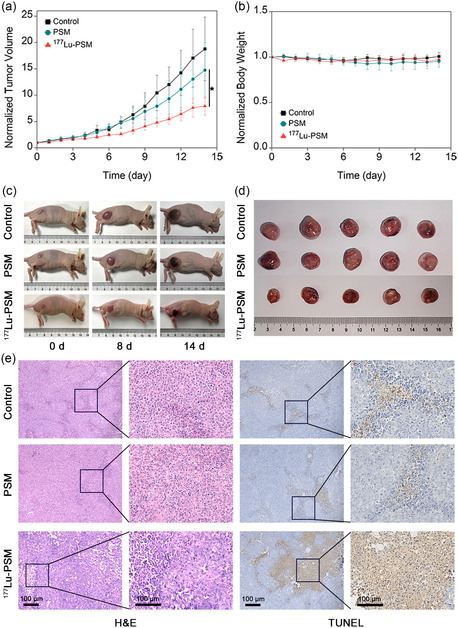
a) Normalized tumor volume and b) body weight of mice after treatment with saline (control), PSM (50 mg kg^−1^), and ^177^Lu‐PSM (100 μCi). c) Photographs of representative mice recorded during the treatment. d) Photographs of tumors after 14 d of treatment. e) H&E and TUNEL staining images of tumor slices of HepG2‐tumor‐bearing mice treated with saline, PSM, and ^177^Lu‐PSM. The error bars represent the SD of five replicates. *P* values were calculated by student's *t*‐test (*P < 0.05, **P < 0.01, and ***P < 0.001).

To investigate the potential antitumor activity of the prepared microspheres, HepG2‐tumor‐bearing mice were treated with normal saline, PSM, and ^177^Lu‐PSM. Subsequently, the temporal variation in the tumor volume and body weight of mice during the treatment were recorded **(** **Figure** **5**a,b **)**. As shown in the figures, tumor progression was obviously inhibited in the group treated with ^177^Lu‐PSM (100 μCi). In addition, negligible variation in the body weights of all three groups was observed. The results suggested that the β‐particles generated from ^177^Lu could effectively kill the tumor cells. In contrast, the tumor sizes increased with a rapid rate for the control group upon a 14‐day treatment. Similarly, the images of animals and their corresponding tumor sites of each group taken on day 14 of treatment (Figure 5c,d) suggested the superior antitumor activity of ^177^Lu‐PSM compared to the control compounds. Furthermore, the antitumor activity of ^177^Lu‐PSM will be better with higher radioactivity according to the results of the cytotoxicity assay.

After treatment, the biodistribution of ^177^Lu was calculated as described earlier. The results shown in Figure S2 (Supporting Information) were consistent with those given above. Almost all radioactivity accumulated well in tumors with a percent ID/g of 131.8 ± 32.0 and little uptake in bone with a percent ID/g of 11.6 ± 4.7. Furthermore, to visualize the structure of tumor cells and investigate the degree to which the cells underwent apoptosis, the tumor tissues of mice treated with saline, PSM, and ^177^Lu‐PSM were stained by H&E and TUNEL. As shown in Figure 5e, significant apoptosis was observed in tumors treated with ^177^Lu‐PSM. In addition, major organs such as the heart, liver, spleen, lung, and kidney were stained by H&E **(**Figure S3, Supporting Information**)**. As seen in the figure, no obvious damage was created by ^177^Lu‐PSM to these organs. This alleviates concerns regarding the potential side effects of radioactive microspheres.

**Figure 6:**
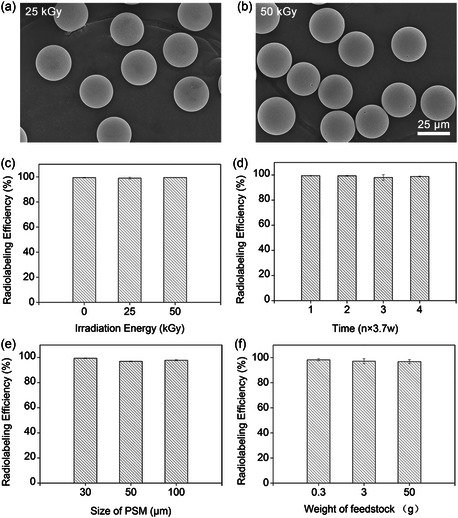
a,b) SEM images of PSM after irradiation‐induced sterilization with total accumulated doses of 25 kGy (a) and 50 kGy (b). c,d) Radiolabeling efficiency of ^177^Lu‐PSM after being exposed to different irradiation (c) and after the accelerated aging test (d). e,f) Radiolabeling efficiency of ^177^Lu‐PSM with different sizes (e) and different weights (f) of feedstock. The error bars represent the SD of three replicates.

As one of the essential measures adopted prior to the administration of interventional devices, γ‐ray‐induced sterilization can be mentioned. In actual courses of sterilization, the applied dose can reach a range of 25–50 kGy to kill the microorganisms. However, γ rays with such doses might have deteriorating effects on the structure of the devices. To investigate the effect of γ‐irradiation on the structure of the prepared microspheres, the samples were exposed to 25 and 50 kGy doses of irradiation. As shown in **Figure** **6**a,b, PSM maintained its spherical structure and smooth surface after being subjected to irradiation. In addition, after being exposed to γ rays, the radiolabeling efficiency of PSM was determined. As illustrated in Figure 6c, the efficiencies after 25 and 50 kGy of irradiation were calculated to be 99.0% ± 0.7% and 99.5% ± 0.1%, respectively, which were almost the same as the control sample (Figure 2a,b). Furthermore, the radiolabeling efficiencies of the samples that were subjected to accelerated aging were investigated. The radiolabeling efficiency did not change noticeably (Figure 6d), such that a value of 98.8% ± 0.4% was calculated after 4 years of equivalent storage on the shelf. Thus, it can be expected that PSM would remain radiolabeled even after a long period of storage, suggesting its potential to be used as a commercial agent for SIRT.

To confirm the universality of the preparation method, PSMs with different sizes were obtained and subjected to functionalization and radiolabeling processes. As shown in Figure 6e, differently sized PSMs exhibited similar and high radiolabeling efficiencies (i.e., 99.4% ± 0.3%, 99.0% ± 0.7%, and 99.5% ± 0.1% for 30, 50, and 100 μm, respectively). In addition, SMs purchased from another provider were used and underwent the same procedure mentioned earlier to produce ^177^Lu‐PSM. The results shown in Figure S4 (Supporting Information) indicated that the microspheres could be phosphonated successfully and lead to a radiolabeling efficiency of 98.2% ± 0.8%. Furthermore, the capability of the method to achieve favorable outcomes while using higher feedstock was explored. As shown in Figure 6f, using a feedstock weight as high as 50 g led to a satisfactory radiolabeling efficiency of 96.8% ± 1.6%. It is worth noting that 50 g of SMs would cost ≈100 dollars, and such a large amount of material can be employed for treating several patients. Thus, the method of production seems to be cost‐effective. Altogether, the method could be applied to SMs of different sources and sizes to prepare ^177^Lu‐PSM with high in vivo stability and low cost. In addition, since the phosphonate group also has strong binding ability with many other metal ions,^[^ ^16^ ^]^ the radiolabeling strategy developed in the current work is expected to be applicable for preparing radioactive microspheres labeled with other metallic radionuclides, for example, ^188^Re, ^90^Y, and ^166^Ho.

## 3 Conclusion

We have successfully developed PSMs through a facile and universal preparation method. The prepared PSM could be effectively labeled with ^177^Lu with a specific activity value higher than those of commercially available radioactive microspheres. It was shown that ^177^Lu‐PSM could kill tumor cells and slow the rate of tumor growth. SPECT/CT images of mice treated with ^177^Lu‐PSM indicated that the product exhibited great in vivo radiolabeling stability and could be used as an agent for imaging‐guided selective internal radiation therapy. In addition, we showed that the preparation method could be used for SMs of different sizes and sources, providing researchers with multiple options depending on the application of interest. More importantly, the results suggested that the product could be mass produced at a low cost. The prepared product could be stored for a long period of time without variation in its properties during the storage period or upon sterilization courses prior to administration. Based on the favorable results obtained in the study, we envision that the prepared radioactive microspheres might be a promising candidate for translation to clinical applications.

## Conflict of Interest

The authors declare no conflict of interest.

## Supporting information

Supplementary Material

## Data Availability

The data that support the findings of this study are available from the corresponding author upon reasonable request.
