# Supplementary material for: Clinically Translatable Phosphonated Silica Microspheres for Selective Internal Radiation Therapy of Hepatocellular Carcinoma
Source: Small Sci. 2023 Aug 22;3(10):2300035. doi: 10.1002/smsc.202300035 (PMC11935814; doi:10.1002/smsc.202300035)
Supplement: Supplementary file 1 — Supplementary Material [file SMSC-3-2300035-s001.pdf]

Supporting Information

Clinically Translatable Phosphonated Silica Microspheres for Selective Internal Radiation Therapy of Hepatocellular Carcinoma

Yi Zhou, Jianxian Ge, Yun Gao, Zhe Yang, Mohammad Javad Afshari, Can Chen, Manran Wu, Lei Chen, Shuwang Wu, Guangxin Duan, Jianfeng Zeng,* Mingyuan Gao**

Center for Molecular Imaging and Nuclear Medicine, State Key Laboratory of Radiation Medicine and Protection, School for Radiological and Interdisciplinary Sciences (RAD-X), Soochow University, Collaborative Innovation Center of Radiological Medicine of Jiangsu Higher Education Institutions, Suzhou 215123, China

Experimental Section

1. Materials

Amino trimethylene phosphonic acid (ATMP) was purchased from Shanghai Meryer Chemical Technology Co., Ltd. β-(3,4-epoxycyclohexane) ethyl trimethoxy silane (EHTMS), tetraethyl orthosilicate (TEOS), ammonium hydroxide (NH_4OH), and dimethyl sulfoxide (DMSO) were purchased from Shanghai Aladdin Biochemical Technology Co., Ltd. Hydrochloric acid was purchased from Chinasun Specialty Products Co., Ltd. Ethanol was purchased from Shanghai Lingfeng Chemical Reagent Co., Ltd. $LuCl_3·6H_2O$ was purchased from Beyotime Biotechnology Co., Ltd. All reagents were of analytical purity and used without further purification. Silica microspheres (SM) were purchased from Suzhou Knowledge & Benefit Sphere Technology Co., Ltd. and Shandong BoNa Biological Technology Group Co., Ltd. $^{177}LuCl_3$ was purchased from Chengdu Qualcomm Isotope Co., Ltd. Milli-Q water (resistivity above 18 MΩ·cm) was used in the experiments.

2. Preparation of phosphonated silica microspheres (PSM).

Phosphonate siloxane was prepared according to the reported method.[1] Briefly, 0.6789 g of ATMP was dissolved in 15 mL of DMSO with stirring at 100°C, and then 0.520 mL of EHTMS was added to the solution. After reacting for 1 h at 100°C under stirring, 300 mg of SM washed 3 times with Milli-Q water after being activated with 3 M HCl at 110°C for 3 h and 1.5 mL of TEOS were added to the solution. The molar ratio of ATMP:EHTMS:TEOS was 1:1:3. The mixture was stirred at 100°C for 48 h, and then the microspheres were filtered and added to a solution of NH_4OH/EtOH (1/100, v/v). After stirring for 12 h at room temperature, the microspheres were filtered and washed with EtOH 3 times and then dried at 140°C. The dry microspheres were washed with Milli-Q water and dried again as mentioned above. Finally, phosphonate silica microspheres (PSM) can be obtained.

3. Preparation of ^{177}Lu-PSM and ^{175}Lu-PSM

For the preparation of ^{177}Lu-PSM, 1 mg of PSM was dispersed in 400 μL 0.2 M sodium acetate buffer solution (pH=4.6), and then the $^{177}LuCl_3$ solution was added. The reaction mixture was shaken in a mechanical shaker at a certain temperature for a period of time. Subsequently, ^{177}Lu-PSM was washed 3 times with saline after being carefully separated from the supernatant. The radioactivity of ^{177}Lu-PSM and the supernatant were measured by a radioactivity meter (FJ-391A4, Beijing Nuclear Instrument Factory).

To characterize ^{177}Lu-PSM, radioactive $^{177}LuCl_3$ was replaced with nonradioactive $^{175}LuCl_3$ to prepare ^{175}Lu-PSM as described above. The morphology of the resultant microspheres was characterized by scanning electron microscopy (SEM, Regulus 8230, Hitachi, Japan), and the particle size and size distribution were determined based on statistical analysis of 100 particles per sample measured with ImageJ. The chemical compositions of the microspheres were analyzed by energy dispersive spectrometry (EDS). The surface element contents of the SM, PSM and ^{175}Lu-PSM were characterized by X-ray photoelectron spectroscopy (XPS, EscaLab 250Xi, Thermo Fisher Scientific, USA).

To optimize the radiolabeling conditions of PSM, the radiolabeling efficiency

under different experimental conditions, such as radiolabeling temperature (25-97°C), shaking time (1-30 min), and PSM concentration (0.05-1 mg/400 μL), was determined. To ensure the reproducibility of the results, each experiment was performed in triplicate.

To determine the maximum specific activity of ^{177}Lu-PSM, different radioactivity of ^{177}Lu were used to radiolabel PSM. The radioactivity per microsphere (R_m) can be estimated using the following equation:

$$R_m = \frac{R}{N} \tag{1}$$

where R is the radioactivity of ^{177}Lu-PSM after radiolabeling, N is the microspheres number of ^{177}Lu-PSM which can be express by

$$N = \frac{m}{\rho \cdot \frac{4}{3}\pi r^3} \tag{2}$$

where m is mass of the ^{177}Lu-PSM, ρ and r are density and radius of the PSM.

4. Radiolabeling stability of ^{177}Lu-PSM *in vitro*

To evaluate the radiolabeling stability of ^{177}Lu-PSM *in vitro*, ^{177}Lu-PSM was incubated in 1.5 mL normal saline, phosphate buffered saline (PBS) and 10% fetal bovine serum (FBS) at room temperature. The activity of the supernatant and total mixture was measured up to 192 h by a gamma counter (LB2111, Berthold, Germany). Each experiment was performed in triplicate.

5. Cytotoxicity assay

HepG2 cells were selected to evaluate the biosafety of PSM and the killing effect of ^{177}Lu-PSM. The relative cell viability was measured by Cell Counting Kit-8 (CCK-8). Specifically, HepG2 cells were added to a 96-well microplate, and each well contained 100 μL of DMEM (including 10% FBS and 1% antibiotic) and then incubated in a cell culture incubator that provided a suitable environment (37°C, 5% CO_2) for 12 h to adhere. Then, the cells were treated with different concentrations of PSM (0, 31.25, 62.5, 125, 250, 500 μg/mL) for 24 h. After co-incubation, the PSM were washed off with PBS. Subsequently, 100 μL of DMEM containing 10% CCK-8 was added to each well for 2 h, and then the absorbance at 450 nm was measured by a microplate reader (iMark, Bio-Rad, USA). To compare the killing effect of free ^{177}Lu and ^{177}Lu-PSM, HepG2 cells were incubated with different radioactivity levels of free ^{177}Lu and ^{177}Lu-

PSM (0, 0.78, 1.56, 3.13, 6.25, 12.5, 25, 50 μCi, 500 μg/mL) for 24 h or 48 h. Each experiment was performed in quadruplicate.

6. Immunofluorescence

HepG2 cells were incubated for 12 h to adhere to the slides and then treated with PSM (0.5 mg) and ^{177}Lu-PSM (0.5 mg, 100 μCi) in a cell culture incubator for 12 h. After co-incubation, the microspheres were washed off with PBS. The cells were fixed with 4% paraformaldehyde for 15 min at 4°C, followed by treatment with Triton X-100 (0.2%) for 10 min at room temperature to improve the permeability of the cell membrane. Then, the HepG2 cells were incubated with mouse anti-γH2A.x antibodies overnight at 4°C after being blocked with 1% bovine serum albumin (BSA) for 1 h at room temperature. Afterward, the HepG2 cells were washed with PBS and incubated with the secondary antibody for 1 h at 37°C. After washing with PBS, the HepG2 cells were treated with Hoechst for 5 min, and then fluorescent images were obtained by a fluorescence microscope.

7. SPECT/CT imaging and radiolabeling stability of ^{177}Lu-PSM *in vivo*

All animal experimental protocols were approved by the Institutional Animal Care and Use Committee of Soochow University (approval number: 202206A0909) and performed in compliance with the National Guide for Care and Use of Laboratory Animals. Six-week-old male nude mice weighing approximately 20 g were purchased from Changzhou Cavins Animal Co., Ltd. The HepG2 tumor-bearing model was established by injecting HepG2 cells subcutaneously. When the volume of tumors grew to 100 mm^3, the HepG2 tumor-bearing mice were intratumorally injected with free ^{177}Lu or ^{177}Lu-PSM with radioactivity of 700 μCi. After injection, SPECT/CT images were obtained by microSPECT/CT (MILabs, U-SPECT$^+$, the Netherlands) at 10 min, 2 h, 8 h, 1 d, 2 d, 4 d, 7 d and 14 d. When SPECT/CT imaging had been accomplished, the HepG2 tumor-bearing mice were sacrificed, and the tumor and main organs were collected to measure the weight and radioactivity for biodistribution.

8. Radiotherapy of tumor and biodistribution *in vivo*

HepG2 tumor-bearing mice were prepared as described above to check the antitumor ability of ^{177}Lu-PSM when used for SIRT. The mice were randomly divided

into 3 groups (n=5) when the volume of tumors grew to 50 mm^3 and treated intratumorally with saline, PSM (50 mg/kg) and ^{177}Lu-PSM (100 μCi, 50 mg/kg). The body weight and the tumor volume were recorded every day to evaluate the antitumor ability during 14 d of treatment, with photographs of mice recorded every two days. After treatment, the mice were sacrificed, and the blood, tumor and main organs were collected to measure the weight and radioactivity for biodistribution. Subsequently, the tumor and organs were soaked in 4% paraformaldehyde solution to make slices. Hematoxylin and eosin (H&E) staining was carried out to confirm whether possible damage to normal organs may be caused by treatment. In particular, terminal deoxynucleotidyl transferase dUTP nick end labeling (TUNEL) staining of tumor slices was carried out to check the apoptosis of tumor cells after 14 d of treatment.

9. Possibility of clinical transformation of PSM

As a kind of interventional device for SIRT, excellent stability of physicochemical properties is necessary. PSM must tolerate the damage of irradiation sterilization and have a long shelf life before being used. To evaluate the stability of PSM, PSM were sterilized by gamma rays with total accumulated doses of 25 kGy and 50 kGy before being labeled. In addition, the accelerated aging test was used to test the shelf life of PSMs. As studies have shown, at an aging temperature of 60°C, 3.7 weeks is equivalent to 1 year on the shelf at room temperature.[2] After irradiation sterilization and the accelerated aging test, PSM were characterized by SEM and used to prepare ^{177}Lu-PSM as described above. SEM was used to confirm any structural damage to PSM, and ^{177}Lu was used to radiolabel PSM to evaluate the stability of PSM. Furthermore, to prove whether the phosphonate functionalization method was suitable for silica microspheres with different sizes, the radiolabeling efficiency of PSM with sizes of 30, 50, and 100 μm was determined. Finally, the possibility of mass production was assessed under feedstock up to 50 g with phosphonate functionalization and radiolabeling conducted as mentioned above. To ensure reproducibility of the results, each experiment was performed in triplicate.

10. Statistical analysis

All data were represented as mean ± standard deviation (SD) unless specified

otherwise (n=3 or 5, see details in the Supporting Information). Statistical significance between two groups was assessed using Student's t-test and the significance level was set at a value of 0.05. The data were indicated as *P <0.05, **P<0.01 and ***P<0.001. The statistical analysis was performed using GraphPad.

Supplementary Results

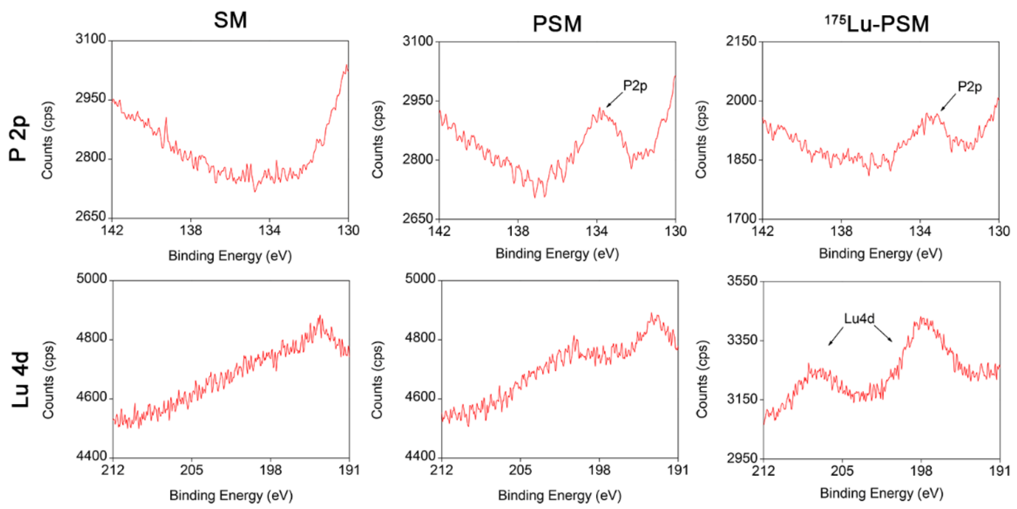

Figure S1. High-resolution XPS results (P 2p and Lu 4d) of SM, PSM and ^{175}Lu-PSM.

Table S1. The element content of SM, PSM and ^{175}Lu-PSM calculated by XPS.

Atomic (%)	SM	PSM	^{175}Lu-PSM
Si	31.55	30.09	30.23
O	61.67	58.09	58.85
C	6.08	10.70	9.92
N	0.71	0.99	0.77
P	/	0.14	0.13
Lu	/	/	0.10

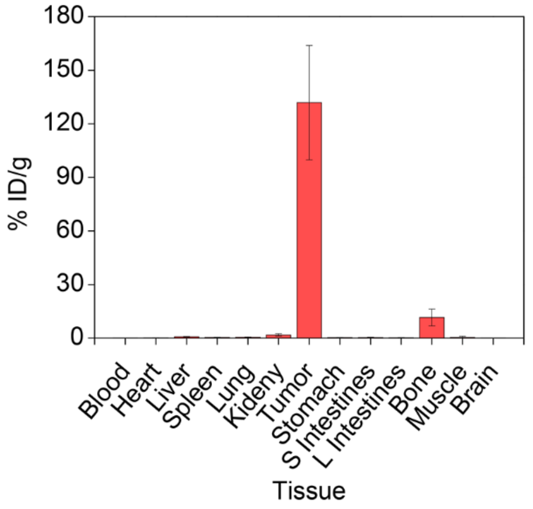

Figure S2. Biodistribution of ^{177}Lu obtained 14 d after ^{177}Lu-PSM treatment. The error bars represent the SD of five replicates.

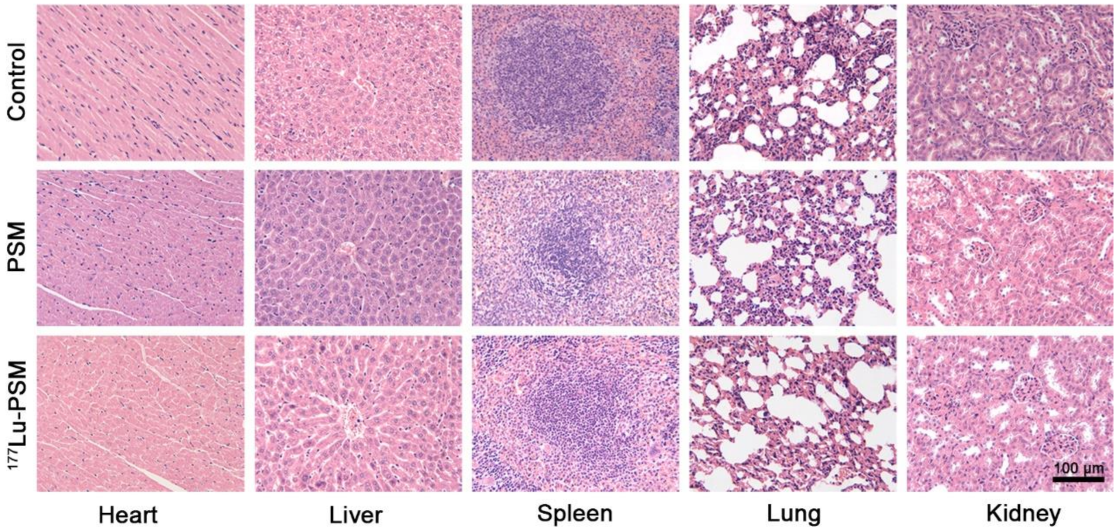

Figure S3. H&E staining of main organs (heart, liver, spleen, lung, kidney) from HepG2 tumor-bearing mice treated by saline (control), PSM (50 mg/kg), ^{177}Lu-PSM (100 μCi, 50 mg/kg).

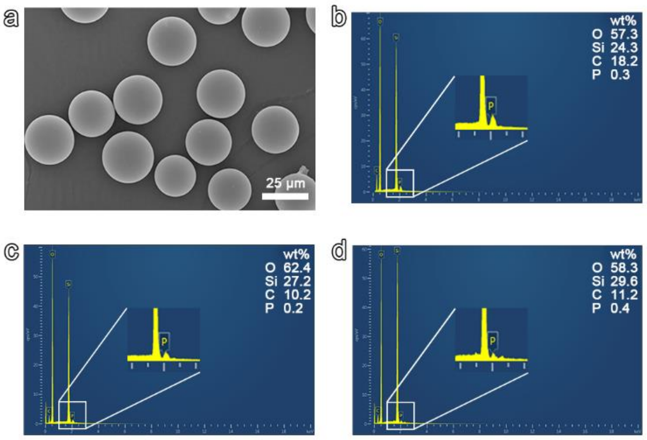

Figure S4. (a) SEM images of PSM. The EDX spectra of PSM with different weight of feedstock: 0.3 g (b), 3 g (c), 50 g (d).

References

[1] W. Li, C. H. Shen, S. J. Gao, S. S. Yin, H. L. Li, *Solid State Ionics* **2016**, *287*, 1.

[2] G. S. Clark, Shelf Life of Medical Devices, http://www.fda.gov/regulatory-information/search-fda-guidance-documents/shelf-life-medical-devices, accessed: April, **1991**.
